# Mobilization of Antibiotic Resistance: Are Current Approaches for Colocalizing Resistomes and Mobilomes Useful?

**DOI:** 10.3389/fmicb.2020.01376

**Published:** 2020-06-30

**Authors:** Ilya B. Slizovskiy, Kingshuk Mukherjee, Christopher J. Dean, Christina Boucher, Noelle R. Noyes

**Affiliations:** ^1^Food-Centric Corridor, Infectious Disease Laboratory, Department of Veterinary Population Medicine, College of Veterinary Medicine, University of Minnesota, St. Paul, MN, United States; ^2^Department of Computer and Information Science and Engineering, The Herbert Wertheim College of Engineering, University of Florida, Gainesville, FL, United States

**Keywords:** antimicrobial resistance, mobile genetic elements, colocalization, assembly, network analysis

## Abstract

Antimicrobial resistance (AMR) poses a global human and animal health threat, and predicting AMR persistence and transmission remains an intractable challenge. Shotgun metagenomic sequencing can help overcome this by enabling characterization of AMR genes within all bacterial taxa, most of which are uncultivatable in laboratory settings. Shotgun sequencing, therefore, provides a more comprehensive glance at AMR “potential” within samples, i.e., the “resistome.” However, the risk inherent within a given resistome is predicated on the genomic context of various AMR genes, including their presence within mobile genetic elements (MGEs). Therefore, resistome risk stratification can be advanced if AMR profiles are considered in light of the flanking mobilizable genomic milieu (e.g., plasmids, integrative conjugative elements (ICEs), phages, and other MGEs). Because such mediators of horizontal gene transfer (HGT) are involved in uptake by pathogens, investigators are increasingly interested in characterizing that resistome fraction in genomic proximity to HGT mediators, i.e., the “mobilome”; we term this “colocalization.” We explored the utility of common colocalization approaches using alignment- and assembly-based techniques, on clinical (human) and agricultural (cattle) fecal metagenomes, obtained from antimicrobial use trials. Ordination revealed that tulathromycin-treated cattle experienced a shift in ICE and plasmid composition versus untreated animals, though the resistome was unaffected during the monitoring period. Contrarily, the human resistome and mobilome composition both shifted shortly after antimicrobial administration, though this rebounded to pre-treatment status. Bayesian networks revealed statistical AMR-MGE co-occurrence in 19 and 2% of edges from the cattle and human networks, respectively, suggesting a putatively greater mobility potential of AMR in cattle feces. Conversely, using Mobility Index (MI) and overlap analysis, abundance of *de novo*-assembled contigs supporting resistomes flanked by MGE increased shortly post-exposure within human metagenomes, though > 40 days after peak dose such contigs were rare (∼2%). MI was not substantially altered by antimicrobial exposure across all cattle metagenomes, ranging 0.5–4.0%. We highlight that current alignment- and assembly-based methods estimating resistome mobility yield contradictory and incomplete results, likely constrained by approach-specific data inputs, and bioinformatic limitations. We discuss recent laboratory and computational advancements that may enhance resistome risk analysis in clinical, regulatory, and commercial applications.

## Introduction

Antimicrobial resistance (AMR) is currently among the most urgent issues facing human and animal health (Roca et al., 2015; de Kraker et al., 2016). AMR arises from the capacity of bacteria to resist antimicrobial drugs through several genetic mechanisms. These mechanisms are complex, and the means by which bacteria acquire these mechanisms are correspondingly diverse. The use of high-throughput sequencing (HTS) to analyze the basis of AMR across bacterial taxa is generating an even deeper appreciation for the microbial ecological complexity of AMR. By taking a metagenomics approach, scientists can describe and semi-quantitate the resistome, i.e., all detectable AMR genes within a given sample. This approach has demonstrated that resistomes are ubiquitous, even in samples with little direct selective pressure (Hughes and Andersson, 2017). In some cases, environments with low anthropogenic influence contain more diverse resistomes than environments with antimicrobial drug residues, for example. These results suggest a need to characterize resistomes using more nuanced methods and metrics (Hu et al., 2013; Martínez et al., 2015; Lanza et al., 2018). Under this perspective, the resistome itself is a hazard, and different resistome constellations confer various levels of risk to public, human, or animal health. The need to discriminate the risk level of various resistomes is especially salient for purposes of food safety and public health risk assessment (Gillings, 2013; Millan, 2018).

One component of evaluating resistome risk is the need to understand the inherent risk level of a given AMR gene. In some cases, these varying risk levels can be easy to discern by most clinicians, regulatory agencies, and scientists. For example, AMR genes that confer resistance to last-resort antimicrobial drugs carry higher risk than AMR genes that confer resistance to antimicrobial drugs that cannot be used in human patients. But the inherent risk level of a given AMR gene is only a small piece of the overall risk picture. In fact, the genomic context of each AMR gene is often more important than the identity and quantity of the AMR genes themselves. The criticality of genomic context arises from the ability of bacteria to propagate AMR genes via several genetic mechanisms, including asexual reproduction and horizontal gene transfer (HGT). The latter mechanism plays an especially important role in the microbial ecology of AMR because it allows distantly related bacteria to exchange AMR genes. Rates of HGT have been shown to increase under salient environmental conditions, and HGT enables AMR gene transfer between pathogens and non-pathogens (Forsberg et al., 2012; Qin et al., 2015; von Wintersdorff et al., 2016). Furthermore, many HGT mechanisms support the packaging and transfer of *sets* of genes, meaning that bacteria can exchange several AMR genes in a single transfer event. The specifics of HGT are highly variable and difficult to predict due to their complexity. The propensity for two bacteria to exchange AMR genes via HGT will depend on several factors, including the identity and genomic composition of the donor and recipient bacteria; the type of HGT mechanism involved; and the environment surrounding the bacteria at any given time. Due to this complexity, a comprehensive view of HGT and AMR genes across all bacteria would be highly relevant and indeed necessary to fully appreciate the clinical and epidemiologic risks intrinsic to the mobilization and acquisition of AMR genes in bacterial populations (Beceiro et al., 2013; Partridge et al., 2018).

Recently, several studies have reported the results of resistome and mobilome analysis, where mobilome is defined as all detectable HGT elements within a given metagenomic dataset, including plasmids, integrative conjugative elements (ICE), transposons, and insertional repeat sequences. These studies have yielded insights into potential connections between AMR ecology and HGT potential (Pal et al., 2015; Che et al., 2019; Ju et al., 2019; Li et al., 2019; Yin et al., 2019). The bioinformatics methods needed to perform mobilome analysis are similar to those needed for resistome analysis, and include a database of reference sequences, a suitable bioinformatic approach, and appropriate statistics. As with AMR genes, a myriad of databases exists for mobile genetic elements (MGEs) involved in HGT (Siguier et al., 2006; Leplae et al., 2010; Bi et al., 2012; Carattoli et al., 2014; Arndt et al., 2016). There is also one dedicated bioinformatics pipeline recently published to support resistome-mobilome analysis (Oh et al., 2018). However, the usability and robustness of these resources to support resistome-mobilome analysis on short-read shotgun metagenomic data has not yet been described. Furthermore, different bioinformatic and statistical approaches may generate orthogonal or complementary insights from the same metagenomic data. For example, one approach may be better suited to identifying broad resistome-mobilome patterns, while another may complement this high-level information by identifying associations between specific AMR genes and specific MGEs. On the other hand, if two approaches yield conflicting information about the same data, this may indicate that one or both methods have surpassed their limit to accurately analyze the data. Improving our understanding of the benefits, limitations, synergies, and inconsistencies of these common approaches would advance resistome-mobilome analysis while also highlighting gaps requiring further methodological advancement.

The need to evaluate current resistome-mobilome approaches has reached a critical threshold due to the combination of: increasing generation and availability of short-read metagenomic data; expanding use of short-read metagenomic data for regulatory and surveillance activities; and increasing scientific awareness of the need for more nuanced characterization of resistome risk. Therefore, the goal of this study was to apply, compare and evaluate the most common bioinformatic and statistical approaches for performing resistome-mobilome analysis on shotgun metagenomic data. To do this, we used two publicly available datasets, both alignment- and assembly-based approaches, and several common statistical techniques.

## Materials and Methods

### Study Overview

**FIGURE 1:**
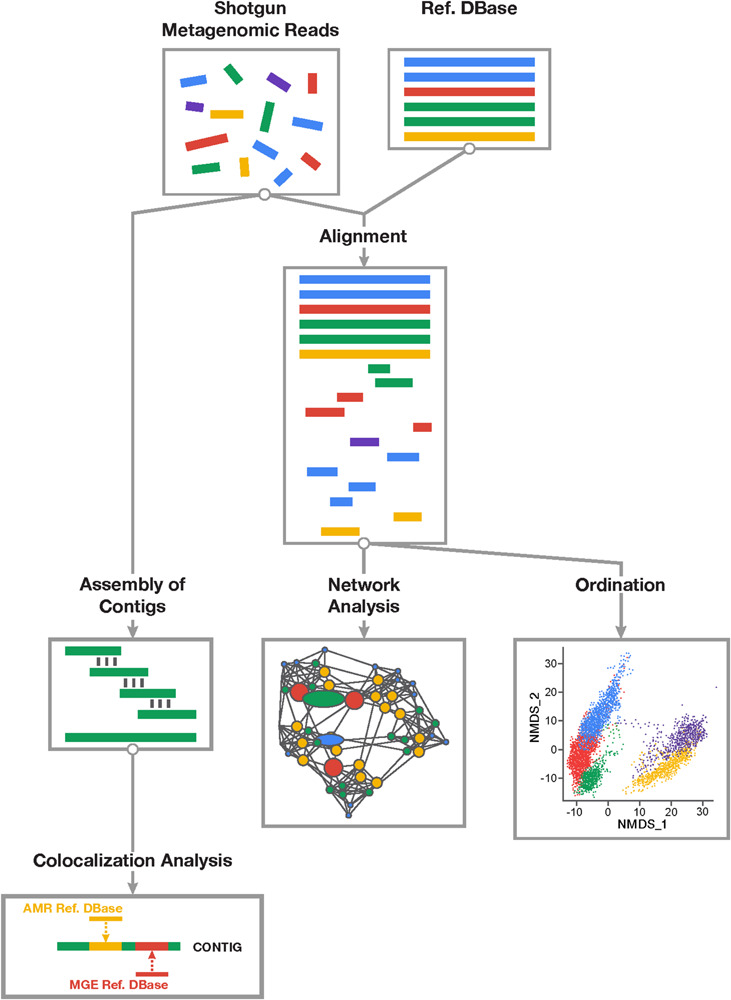
Overview of study workflow involved in resistome-mobilome analysis of shotgun metagenomic datasets to characterize resistome mobility potential. Short reads derived from shotgun metagenomic sequencing platforms can be assessed using two general approaches: alignment-based and assembly based. Alignment-based (right division) approach involves mapping of sequencing reads to reference databases of antimicrobial resistance genes and mobile genetic elements. Statistical colocalization can then be achieved by constructing predictive co-occurrence networks as well as non-parametric ordinations using count matrices of positive gene alignments to resistance or mobility reference sequences. Assembly based approach (left division) involves *de novo* reconstruction of contiguous sequences from short metagenomic read using existing assembler algorithms and computational pipelines. Resulting contigs can be mapped to both resistance and mobile genetic element reference databases and those contigs supporting co-occurring sequences can be detected and quantified in samples.

Using a general framework described in Figure 1, we evaluated the use of archetypal open-source alignment- and assembly-based bioinformatic approaches, as well as data reduction and network statistical methods to perform resistome-mobilome colocalization analysis (Table 1). To reflect the most typical use cases for colocalization analysis, where possible we performed all procedures using default parameters. We also utilized thresholds most reported in the resistome-mobilome literature (Table 1).

**TABLE 1:** Overview and basic characteristics of AMR-MGE colocalization analysis using two general approaches: alignment and assembly.

**Approach**	**Alignment-based**			**Assembly-based**	
**Method**	**Ordinatation**		**Network analysis**	***De novo* assembly**	**Resistome risk pipeline**
Operation	NMDS	• Biplot Analysis	• Bayesian Network: Directed Acyclic Graph (DAG)	• Linear DNA Metagenomic Assembly	• Analysis Pipeline using IDBA-UA Assembly
Program	Vegan v2.5-5	• BNLearn v4.4.1	• MetaSPAdes	• MetaCompare	
Input Data	• AMR/MGE accession count matrix filtered for sparse genes • Normalized (CSS) • Transformed (Hellinger)	• AMR/MGE accession counts, metadata variables (continuous or categorical) • Blacklisting non-sensical network relationships (arcs)	• Trimmed pair-end FASTQ files	• Trimmed pair-end FASTQ files	
Database	Pipeline: *AMRPlusPlus* v 1.0.1 Plasmids: PlasmidFinder v 2.1 Prophage and other MGE : ACLAME v 0.4	AMR: MEGARes v 1.0 ICE: ICEBerg v 2.0	MEGARes v 1.0, PlasmidFinder v 2.1, ICEBerg v 2.0, ACLAME v 0.4	CARD, ACLAME v 0.4, PATRIC v 3.6.2	
Analytical Parameters	• K (stress) < 0.2, good 2D representation in reduced dimensions • Anosim R > 0.75, well separated • Anosim *R* = 0.5, moderate overlap • Anosim *R* < 0.25, significant overlap	• M^2^ test statistic • *P* < 0.05	• Learning algorithm: Hill climb • BN Classifier: Naive Bayes • Conditional independence test: hybrid modeling • Arc blacklisting • Bootstraps: 1000 • Empirical threshold frequency: 0.70	• Default assembly parameters • BLAST to MGE or AMR databases for contigs >800 bp of a continuous stretch with >90% sequence identity and *E*-value < 1 × 10^–10^	• Default assembly parameters • BLAST alignment to MGE or AMR based on defaults
Colocalization	• 2D spatial visualization of AMR/MGE genes (at any ontologic level) according to a metadata variable	• Overlay of AMR and MGE NMDS plots showing co-occurrence according to a metadata variable	• Predictive network of AMR and MGE gene co-occurrence and visualization of transitivity and centrality of critical genes/groups	• Mobility index (MI): Number of contigs containing an AMR gene type and MGE gene divided by total number of contigs containing an AMR gene type in a sample	• Risk score • 3D Risk visualization
Method Ref.	• Ordination: (Oksanen, 2019) • CSS Normalization: (Paulson et al., 2013) • Hellinger Transformation: (Legendre and Gallagher, 2001)	• Bayes network analysis: (Scutari, 2010)	• Assembly defaults: (Nurk et al., 2017)	• Assembly defaults: (Peng et al., 2012) • Pipeline default: (Oh et al., 2018)	

*Basic characteristics of metagenomic colocalization are described.*

### Test Data Overview

In the current work, co-occurrence analysis was performed using data from two previously published studies that performed metagenomic Illumina sequencing of DNA from fecal samples. In both studies, feces were collected from healthy individuals before and after antimicrobial drug exposure. Both studies evaluated resistome dynamics over time. To represent a range of metagenomic samples, we chose one study conducted in humans (Palleja et al., 2018) and another conducted in U.S. feedlot cattle (Doster et al., 2018) (referred to as “human” and “cattle,” respectively). In the human trial, a 4-day combination course of parenteral last-resort antimicrobials (meropenem, gentamicin, and vancomycin) was given to 12 individuals who were sampled at five time points (Day 0-pre-treatment, Day 4-last day of treatment, and Days 8-, 42-, 180-post-treatment). The cattle trial involved a single course of parenteral tulathromycin given to 15 individuals (treated) and another 15 individuals in a nearby pen who were kept untreated. For both groups of cattle, fecal samples from individual cattle were obtained at two time points (Day 0-“pre-treatment” and Day 11-“post-treatment”).

### Nucleotide Sequence Data

Sequence and metadata for the samples used in this study are publicly available under BioProject accessions PRJEB20800 (human; 2 × 100 bp, *n* = 55) and PRJNA309291 (cattle; 2 × 125 bp, *n* = 60). Both studies reported formal comparison of sequencing depth and other experimental metadata variables between sample groups, and concluded that there was no evidence of sequencing or experimental bias impacting the comparison of resistomes across treatments or individuals. To ensure equal treatment and filtering of reads for purposes of our analysis, both datasets were processed using default parameters of the AMRPlusPlus (v1.0.1) bioinformatic pipeline (Lakin et al., 2017), which integrates sequence trimming and quality filtering using Trimmomatic (Bolger et al., 2014)**;** host subtraction after aligning samples to either *Bos taurus* (UMD3.1) or *Homo sapiens* (version hg19) reference genomes using the Burrows-Wheeler-Aligner (BWA) software (Li, 2013); and delimitation of non-host reads using SamTools (Li et al., 2009).

### Databases Used in Analysis

Both alignment- and assembly-based approaches require the use of databases for identification of target sequences. For resistome analysis using alignment-based (referred to as “Alignment”) and assembly-based (referred to as “Assembly+BLAST”) techniques, we used MEGARes v 1.0.1 (Lakin et al., 2017) as the reference database. Any MEGARes accessions that included the addendum “*RequiresSNPConfirmation*” in the header were removed from analysis, as additional confirmatory assessments would be needed to ensure positive detection of these genes. Reference mobilome genes used for “Alignment” and “Assembly+BLAST” were derived from ICEberg v 2.0.0 (Liu M. et al., 2019), PlasmidFinder v 2.0.2 (Carattoli et al., 2014), and ACLAME v 0.4 (Leplae et al., 2010) databases; from PlasmidFinder, only the accessions for gram-positive and *Enterobacteriaceae* were included. For the “Alignment” and “Assembly+BLAST” approaches, all MEGARes and MGE accessions were concatenated into a single FASTA file, resulting in a database size of 129,355 AMR, plasmid, integrative conjugative element, mobilizable element, prophage, and other virion accessions.

For the second assembly-based technique using the risk-oriented *MetaCompare* pipeline (Oh et al., 2018), we utilized the reference databases included with the default implementation of *MetaCompare*, i.e., CARD for AMR gene reference accessions (McArthur et al., 2013) and ACLAME for MGE reference accessions (Leplae et al., 2010). This pipeline also uses PATRIC v 3.6.2 reference accessions (Wattam et al., 2014) to identify potential pathogens within the metagenomic data in order to generate its resistome risk score. To maintain fidelity of the *MetaCompare* pipeline, we included only the CARD and ACLAME reference databases in our analysis of the *MetaCompare* assembly results, and databases were downloaded as part of the default *MetaCompare* installation, which is dated as 03.29.2018.

### Resistome-Mobilome Colocalization Using Alignment-Based Approaches

Metagenomic reads from the human and cattle samples were analyzed for their resistome and mobilome content using the AmrPlusPlus pipeline, which identifies alignments to a pre-specified nucleotide database of accessions using BWA, and then parses the resulting SAM file to count alignments and calculate the gene fraction for each identified accession. Gene fraction is defined as the proportion of nucleotides within a given reference accession that are aligned by at least one sequence read. To avoid false positive detection of AMR and MGE genes, we used the default gene fraction value of AMRPlusPlus, i.e., 80% (Lakin et al., 2017). This default parameter is consistently used by recent studies deploying AMRPlusPlus (Rowe and Winn, 2018; Berglund et al., 2019; Liu J. et al., 2019; Rovira et al., 2019). The output of AMRPlusPlus is a matrix containing counts of aligned reads for each accession identified with at least 80% gene fraction. For resistance genes, AMRPlusPlus also integrates an ontology, and each MEGARes accession is classified within this ontology at the group, mechanism and class levels (Lakin et al., 2017). Therefore, we aggregated accession-level counts to each of these levels within the ontology (see Supplementary Datasheets 1, 2 for a per-sample summary of alignment counts to AMR gene groups).

To broadly compare the resistome and mobilome between the two sample sets (“human” versus “cattle”), we visualized the raw abundance of AMR genes and MGEs at various levels of ontology and classification (i.e., gene, group, mechanism, and class), using KronaTools (Ondov et al., 2011). Then, prior to performing alignment-based statistical analyses, sparse features were removed from all samples (i.e., features present in fewer than 2 samples and with fewer than 2 hits). Due to differences in sequencing depth across samples within a study, all counts were normalized using cumulative sum scaling (CSS) as implemented in the package “metagenomeSeq” (Paulson et al., 2013). Numerous normalization techniques have been deployed in gene-abundance analysis, and all have variable performance in terms of false-positive rate and discovery of differentially abundant genes (Gloor et al., 2017; Weiss et al., 2017). Previous systematic evaluations demonstrate that CSS performs well in appropriately sampled metagenomes (Pereira et al., 2018). After CSS normalization, counts were aggregated at each level of hierarchical organization (i.e., group, mechanism, class, trait). Normalized count matrices for each dataset (i.e., human and cattle) were then used for ordination and network analysis.

Ordination methods have been applied to count matrices derived from shotgun sequence data of microbiomes and resistomes (McMurdie and Holmes, 2013; Noyes et al., 2016; Quince et al., 2017; Tessler et al., 2017; Li et al., 2019). We deployed this approach in the simultaneous exploration of resistome and mobilome compositional changes, especially as a function of specific metadata variables. Ordination of the resistome and mobilome was performed for human and cattle datasets based on Euclidean dissimilarity indices computed from Hellinger-transformed (Legendre and Gallagher, 2001) cumulative sum scaling-normalized (Paulson et al., 2013) counts using the *vegdist* and *decostand* function in “Vegan” (Oksanen, 2019). *metaMDS* was used to perform non-metric multidimensional scaling (NMDS) to arrive at a stable ordination solution. If necessary, the number of ordination dimensions were iteratively increased until a stress value < 0.05 was achieved. Analysis of similarity (ANOSIM) was utilized as an “omnibus” test (Clarke, 1993) to assess significance of separation between sample groups over sampling timepoints, at the mechanism level for AMR genes, and for plasmid, ICE, and prophage genes in both study sets. In a similar fashion, to further investigate any differences in resistome and mobilome composition over time in the human and cattle datasets, we employed permutational multivariate analysis of variance (PERMANOVA) on Euclidean distance matrices, according to (Anderson and Walsh, 2013), using the *adonis* function in “Vegan.” As both ANOSIM and PERMANOVA tests are susceptible to dispersion heterogeneity and therefore may confound between-group variation with within-group variation, we calculated beta-dispersion associated with all group centroids using the *betadisper* function, and we explored the multivariate homogeneity of group dispersion using *permutest.* All permutive methods used 999 permutations and *P* < 0.05 was used as the significance threshold for all test statistics. To measure the degree of correlation between the resistome and mobilome (i.e., the correlation between AMR genes and MGEs) in each dataset over time, Procrustes analysis was used to maximize similarity between NMDS ordinations of AMR and MGE features, and the *protest* function in “Vegan” was used to obtain a correlation value and significance test statistic (*M*^2^). For the human study, Procrustes analysis of the co-occurring resistome and mobilome was performed across all samples for pre-treatment, peak treatment, and post treatment time points. Procrustes analysis for samples in the cattle study were performed by treatment group, and by time within each treatment group. Procrustes analysis has been previously applied in a number of studies including the characterization of resistomes and colocalization of resistant determinants and MGEs (Noyes et al., 2016; Wu et al., 2017, 2018; Zhu et al., 2017; Liao et al., 2019).

While ordination techniques can uncover temporally dynamic correlation between the *overall* composition of AMR genes and MGEs in a set of samples, network analysis can be used to identify associations between specific genomic features (Barberán et al., 2012; Li et al., 2015, 2016; Liu et al., 2015; Tung et al., 2015; Feng et al., 2018; Noyes et al., 2018; Yin et al., 2019). Because of their flexibility, networks have recently been used to identify associations between AMR genes and MGEs (Pal et al., 2015; Hu et al., 2016; McGeachie et al., 2016; Yin et al., 2019). These studies used both non-random co-occurrence networks and Bayesian networks, the latter of which are flexible enough to incorporate diverse data including sample metadata, environmental, microbial, and host factors (Nagarajan et al., 2013; Shafiei et al., 2014; Noyes et al., 2018). Using as inputs the gene taxonomy information, sample metadata, and alignment-derived counts, we constructed Bayesian Networks (BNs) for each dataset to identify edges between AMR genes and MGEs, all aggregated to the mechanism level of genomic ontology. The resulting consensus graphs were displayed using a force-directed visualization algorithm (Jacomy et al., 2014) using GEPHI v 0.9.2 (Bastian et al., 2009). Community structure of the resulting consensus network was discovered using the Louvain modularity class algorithm of Blondel et al. (2008). Input to network analysis consisted of normalized MGE and AMR counts, as well as metadata variables including time and treatment status. Non-sensical temporal associations between samples were blacklisted from the network. The structure of the directed acyclic graph (DAG) was developed using the hill-climbing algorithm as implemented in “bnlearn” within R (Scutari, 2010; Package “bnlearn”, 2019). MGE and AMR counts were modeled with a Gaussian distribution and metadata factors were modeled as binomial variables, using the hybrid model within “bnlearn.” To obtain a stable network, 1000 bootstraps of the input data were specified. The *averaged.network* function was used to construct a consensus network, with an empirical threshold frequency for consensus edges set at 0.70 or more. The resulting DAG was used in maximum likelihood estimation parameter fitting through the *bn.fit* function, as well as network analysis (Scutari, 2010).

### Resistome-Mobilome Colocalization Using Assembly-Based Approaches

*MetaCompare* utilizes the IDBA-UD assembler, which implements a metagenomic-specific assembly algorithm (Peng et al., 2012). MetaSPAdes is another widely used metagenomic-specific assembler (Nurk et al., 2017). To evaluate the impact of assembler choice, we assembled a subset of samples from each dataset using both IDBA-UD (as implemented in *MetaCompare*) and metaSPAdes. MetaSPAdes was run using default parameters, and IDBA-UD was run as implemented in the *MetaCompare* pipeline. Resulting assemblies were evaluated using QUAST v 5.0.2 (Gurevich et al., 2013) with default parameters. Metrics evaluated included N50 and number of contigs > 500 bp. Comparisons were made using a linear mixed model with dataset specified as the random effect with random slope. Statistical significance was evaluated using the *anova* function in R package “lmerTest.”

To parse the metaSPAdes assemblies for resistome-mobilome content, contigs were aligned against the combined MEGARes and MGE database BLAST (Table 1). An *e*-value threshold (<1 × 10^–10^), and sequence identity (>90%) on contigs > 800 bps were used based on common cut-offs described in the literature (Kristiansson et al., 2011; Elbehery et al., 2016; McCall and Xagoraraki, 2018). IDBA-UD contigs were analyzed using the *MetaCompare* pipeline, which aligns PRODIGAL-annotated contigs against CARD, ACLAME and PATRIC using BLAST. *MetaCompare* includes several default cut-offs for parsing BLAST outputs which are applied differently to AMR genes and MGEs. These default settings were used in our analysis to reflect the typical use-case for *MetaCompare* (Oh et al., 2018). AMR genes were identified via BLAST(X) using *e*-value < 1 × 10^–10^, > 60% identity, and minimum alignment length > 25 amino acids, while identification of MGE sequences via BLAST(N) was based on *e*-value < 1 × 10^–10^, > 60% identity, and accessions were to be included if these were associated with > 90% reference sequence coverage.

Once AMR genes and MGEs are identified within assembled contigs, the next step is to identify which contigs contain *both* an AMR gene *and* an MGE; co-occurring placement within a single contig is considered evidence for putative genomic colocalization (Ng et al., 2017; Sáenz et al., 2019; Verma et al., 2019). The *MetaCompare* pipeline performs this type of colocalization and then uses the resultant information to calculate a “risk score” based on the following criteria (in increasing order of risk): Proportion of contigs that contain AMR genes and no MGEs or pathogen-specific sequences; proportion of contigs with both AMR genes and MGEs; proportion of contigs with AMR genes, MGEs and pathogen sequences. We ran *MetaCompare* with default settings and reported the resulting risk score for each sample. To perform comparable analysis with the metaSPAdes-generated contigs, we parsed the BLAST outputs to identify contigs that mapped to at least one AMR gene and one MGE (bioinformatic repository of parsing scripts)^1^; such contigs were considered to contain colocalizations. To confirm the accuracy of this colocalization, we parsed such contigs using BLAST alignment start and stop information. Specifically, we compared the start and stop position of each AMR gene and each MGE within a single contig, and if these positions overlapped, we flagged the contig as containing “overlapping” AMR-MGE alignments. Finally, we calculated a theoretical “mobility index” (MI) for each sample, defined as the fraction of all AMR-harboring contigs that also contained at least one MGE. To evaluate the impact of filtering for AMR-MGE overlap, we reported the raw and overlap-adjusted MIs for each sample.

### Comparison of All Methods

The number of unique AMR gene groups and MGE accessions identified in each sample by each of the three methods was compared after initially filtering for sparseness. The relative abundance of AMR and MGE gene groups identified for all methods and across both datasets were summarized with Venn diagrams constructed in InteractiVenn (Heberle et al., 2015). From each dataset, using the resistome which has a clear ontological hierarchy, we explored in greater depth how each bioinformatic approach (Alignment, *MetaCompare*, and metaSPAdes+BLAST) impacts the total complement of specific genes identified, and therefore would impact resistome-mobilome colocalization. To do this we constructed binary heatmaps using the *heatmap.2* function in the package “gplots” (Warnes et al., 2020).

## Results and Discussion

**FIGURE 2:**
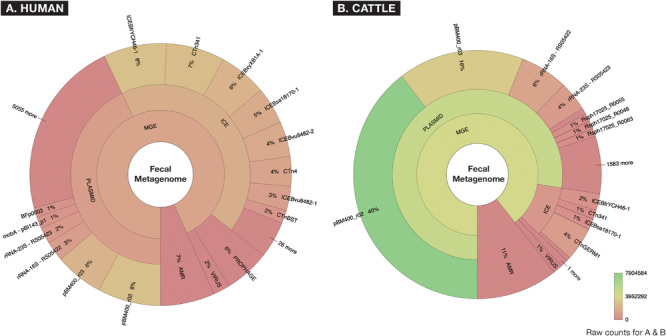
Fecal metagenomes collected from **(A)** human (*n* = 60) and **(B)** beef cattle (*n* = 60) who were parenterally exposed to therapeutic or metaphylactic levels of antimicrobial drugs in controlled trials, were imported from ENA (EMBL-EBI) ERP022986 (Palleja et al., 2018) and SRA (NCBI) PRJNA309391 (Doster et al., 2018), and raw abundance of AMR or MGE accessions were displayed using Krona. Counts are aggregated to taxonomic hierarchies that are shown at the highest level in the center (AMR or MGE) and progress outwardly to lower levels of hierarchy for all MGEs. A hue gradient of red to green is applied to represent a range of raw count frequency, indicating that while human and cattle metagenomes contained similar proportions of AMR accessions, the two metagenomes differed significantly in the constellation of mobile elements (i.e., “mobilomes”) of fecal metagenomes.

After removal of low-quality and host-associated reads, the human and cattle datasets contained ∼1.73 and ∼2.26 billion paired reads [per sample median (range): 31.98 (11.03–69.65) and 38.31 (9.47–68.07) million paired reads], respectively. To characterize the relative abundance of resistome and mobilome features across both datasets, we used raw counts of alignments to reference AMR and MGE databases. These counts indicated that the mobilome comprised a larger proportion of sequence reads than the resistome, in both sample sets (11% of all alignments in the cattle dataset and 7% in the human dataset, Figure 2). However, the mobilome composition of the datasets differed substantially; almost all mobilome alignments in the cattle dataset originated from plasmids, while the human mobilome alignments were distributed relatively evenly between plasmids and ICE (Figure 2). The cattle plasmidome was dominated by alignments to pBM400, a high-molecular-weight plasmid first associated with Bacillus megaterium QM B1551 (Scholle et al., 2003). These high-level differences in mobilome abundance and distribution likely impact the intra-microbial mobility dynamics of AMR genes (Gibson et al., 2015; Bengtsson-Palme et al., 2018).

### Resistome and Mobilome Compositions Differ by Bioinformatic Approach

**FIGURE 3:**
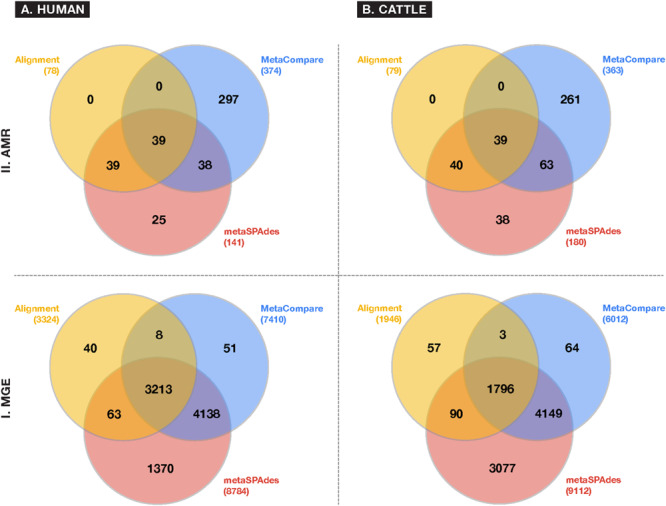
Venn diagrams depicting frequency of overlapping and differing unique gene groups identified in the **(A)** human and **(B)** cattle metagenomic datasets for either **(I)** MGE or **(II)** AMR accessions, using common approaches to colocalize resistomes and mobilomes: Sequence alignment (yellow); direct metagenomic assembly-metaSPAdes (red); and assembly based risk pipeline-*MetaCompare*. Numbers in parenthesis represent the total number of unique gene groups identified by a given approach.

A comparison of resistome and mobilome richness revealed that alignment identified fewer unique AMR groups compared to assembly (Figure 3), in both human and cattle fecal samples. Notably, each assembly method was able to identify 100% of the AMR groups identified by alignment, while alignment did not identify any AMR groups not also detected by the assembly-based approaches. Amongst assembly results, the *MetaCompare* pipeline consistently revealed a greater number of unique AMR groups (human = 374; cattle = 363) relative to metaSPAdes+BLAST (human = 141; cattle = 181). Moreover, of the total number of AMR gene groups identified by each assembly method, 72% (human) and 79% (cattle) of AMR genes identified by *MetaCompare* were not identified by metaSPAdes+BLAST, while 18% (human) and 21% (cattle) of genes identified by metaSPAdes+BLAST were not identified by *MetaCompare*. A similar pattern was observed for the mobilome (Figure 3), with alignment identifying two to fourfold fewer unique gene groups relative to the assembly methods. Notably, a much more notable overlap existed in the capacity of all techniques to identify similar MGE genes, however, metaSPAdes identified more unique MGEs in the human and cattle datasets, while also detecting a majority of MGE groups detected by the other methods.

Several variables likely contributed to the gene richness disparity observed in the data. First, the process of aligning sequence data to reference sequences involves some level of “matching stringency.” While aligners like BWA and BLAST have built-in or default alignment criteria, they are not always suited to metagenomic data and therefore can lead to false-positive identification of AMR genes or MGEs (Haft et al., 2018). Metagenomicists attempt to ameliorate this possibility by employing additional thresholds such as “% sequence identity,” “% length coverage,” statistical measures of homology (e.g., *E*-value), or some combination thereof. However, identity standards or cut-offs are not consistently applied, especially for metagenomic analyses. For example, *MetaCompare* sets gene mapping thresholds for AMR genes at > 60% identity with minimum alignment length of 25 amino acids, and an *E*-value < 1 × 10^–10^. Though metaSPAdes has not been formally benchmarked for AMR genes, previous work utilized a > 90% identity threshold and > 800 bp minimum alignment length to evaluate gene prediction from assemblies (Nurk et al., 2017). A further complication arises when comparing assembly results to alignment results, as tools such as BWA typically utilize a stringent alignment criterion as default, but do not apply a “minimum match length” beyond that of a single read. Because this can create many false positive identifications in metagenomic data, the AMRPlusPlus pipeline applies a gene coverage threshold (i.e., gene fraction) of > 80%, in addition to the default alignment parameters of BWA-MEM. Alignment-based approaches like AMRPlusPlus therefore reduce rates of false-positive inflation, though this is done at the risk of missing divergent, low-abundance, under-characterized, or novel genes (Arango-Argoty et al., 2018). We hypothesize that this strict filtering approach implemented in the alignment-based pipeline likely resulted in loss of detected AMR and MGE group diversity compared to assembly-based results. However, conversely, the assembly-based results may also contain false-positive identifications. It should also be pointed out that applying a gene fraction may inadvertently treat AMR or MGE genes of varying lengths, unequally, thus making within-resistome or -mobilome comparisons difficult. However, this systematic bias is difficult to overcome in current metagenomic practice.

**FIGURE 4:**
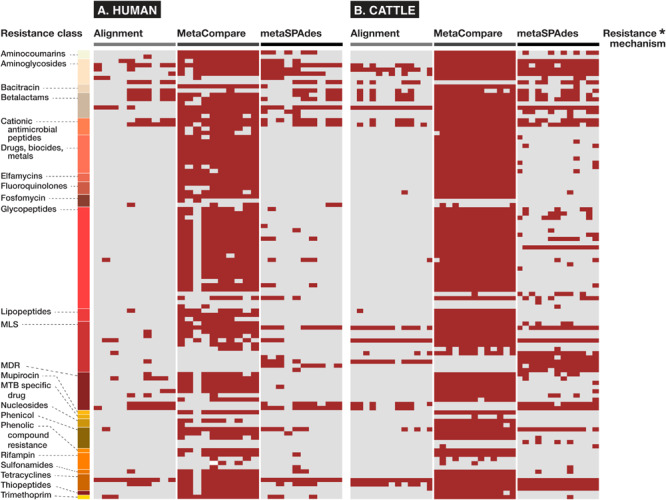
Binary heatmap showing presence (red) or absence (gray) of resistance mechanisms and classes identified in fecal samples of **(A)** human and **(B)** cattle metagenomic datasets, using either alignment, *MetaCompare*, or metaSPAdes bioinformatic approaches. (*Supplementary Datasheet 5 contains a listing of specific resistance mechanism names corresponding to the rows of resistance gene classes).

In addition and related to the issue of gene fraction thresholds in alignment, we note that alignment-based methods disproportionately penalize mapping of gene groups that are characterized by numerous reference database accessions with significant sequence similarity or capacity for cross-resistance to multiple or non-specific compounds. Within the AMR field, this occurs most apparently for resistance to glycopeptide antimicrobials whose resistance sequences have extensive homology (Zeng et al., 2016), betalactamase class of AMR genes (Xavier et al., 2016) for which a single amino acid difference is sufficient to constitute a new “gene” within public databases (Bush, 2018), or general resistance genes which encode molecular machinery connoting resistance to a broad range of substrates (e.g., metals, biocides, etc.) (Pal et al., 2015). For such genes, alignments to short reads are less likely to meet the coverage threshold to be considered as “present” within a given sample. On the other hand, assembly-based methods are more likely to detect these genes, as there is a greater probability that longer stretches of DNA (contigs) facilitate discerned and accurately mapped accessions. The impact of this on resistome analysis, and therefore, any subsequent efforts to establish resistome-mobilome co-occurrence is highly consequential. We demonstrate the unintended effect of these bioinformatic approaches on gene finding by reporting presence/absence distributions for resistance genes at the class and mechanism level using either Alignment, *MetaCompare*, or metaSPAdes+BLAST applied to both the human and cattle datasets (Figure 4). For example, Figure 4 indicates a notable difference in the number of betalactamase resistance mechanisms identified between alignment and assembly-based methods. Within the human and cattle studies, respectively, alignment identified 9 and 8 unique β-lactamase gene groups, whereas *MetaCompare* identified 85 and 67, and metaSPAdes found 23 and 24. Among those gene groups not identified by alignment, the assembly-based approaches identified *bla*_TEM_, *bla*_IMP_, *bla*_VIM_ and *bla*_OXA_ and *bla*_PER_, *bla*_VEB_, *bla*_CMY_. The fact that disparity in discovery rates of these gene groups exists is problematic for several reasons. First, genes including *bla*_TEM_, *bla*_OXA,_ and *bla*_CMY_ connote extended-spectrum betalactamase (ESBL) and AmpC resistance. These genes have emerged as a major global source of AMR in gram-negative pathogens, and have been linked to increased mortality, hospitalization length, healthcare cost, and multi-drug resistance (Schwaber et al., 2006). Second, treatment options for ESBL-containing pathogens are limited and often withheld from common use. Last resort carbapenems are frequently applied to ESBL producing pathogens. It is therefore noteworthy that carbapenemase gene groups, including *bla*_IMP_, *bla*_VIM_, and *bla*_OXA_ were identified by assembly-based methods, but not by alignment in the human and cattle fecal samples. Third, it is well-established that the ESBL genes are frequently disseminated and mobilizable via MGEs, including plasmids, insertional sequences, integrons, and transposons (Singh et al., 2018). Consequently, downstream colocalization efforts using inputs from alignment data may lead to group-by-group bias within results, relative to the other methods.

Lastly, it is important to note that the bioinformatic approaches evaluated in this study rely on different AMR gene databases. While the metaSPAdes+BLAST and Alignment approaches utilized the MEGARes database to identify AMR genes, *MetaCompare* utilized CARD. Across both datasets, *MetaCompare* generated much higher AMR gene richness across all samples relative to metaSPAdes, and this result may be explained by database difference. In addition, *MetaCompare* includes a 25-aa minimum length cut-off for alignments between contigs and AMR accessions in CARD, while the metaSPAdes+BLAST approach applied a minimum length of 800 bp based on previous literature (Nurk et al., 2017). This difference could also account for the greater number of AMR gene groups identified by *MetaCompare* versus metaSPAdes.

As with AMR richness, we identified differences in richness of MGEs across all samples depending on bioinformatic technique. It is likely that the same factors that resulted in disparity in the resistome results (discussed above) also impacted divergences in mobilome results. However, the stark disparity in unique MGE accessions discovered by the two assembly-based methods (Figure 3) can largely be explained by the differences in the associated reference databases used to find genes via each technique. MGEs in IDBA assemblies were identified using ACLAME only (as part of the *MetaCompare* pipeline), while MGEs in metaSPAdes assemblies were identified using ICEberg, PlasmidFinder, and ACLAME. In addition, *MetaCompare* applies a 90% gene fraction cutoff in identifying MGEs, which likely results in dramatic filtering of alignments, especially given that many MGEs are several kilobases (or even megabases) in length and the majority of contigs are <2,000 bp. By contrast, most BLAST parsing approaches in the metagenomic literature do not include a gene fraction cutoff, and therefore the metaSPAdes+BLAST results did not include this criterion.

These results demonstrate that even when using default parameters, current bioinformatic approaches generate different resistome and mobilome profiles for the same source data. As with many bioinformatic applications, choice of reference database and filtering criteria greatly influence the number and diversity of features detected in the metagenomic data. Because colocalization analysis follows basic resistome and mobilome characterization, the differences in detected resistome and mobilome richness likely lead to differences in colocalization results between the approaches.

### Alignment: Temporal Interactions Between Mobilome and Resistome Based on Ordination Analysis

**FIGURE 5:**
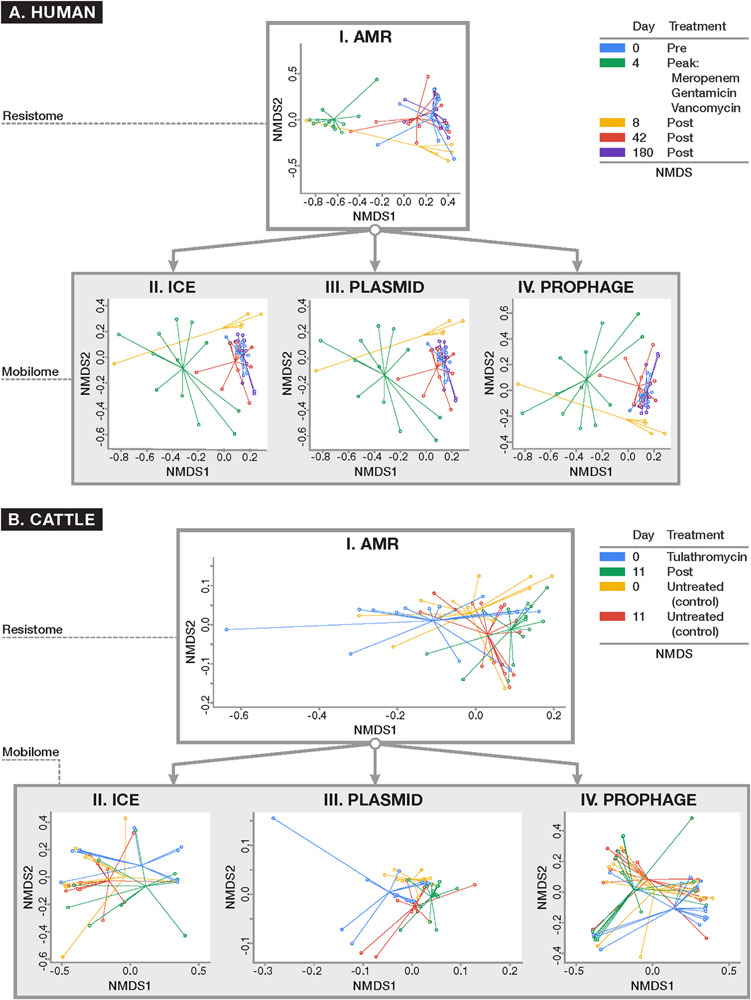
Ordination plots comparing **(I)** resistome composition and **(II–IV)** mobilome composition using non-metric multidimensional scaling (NMDS) for **(A)** human and **(B)** cattle metagenomic datasets. All ordinations were based on Euclidean distances derived from Hellinger-transformed normalized counts of positive alignments. Results of resistome and mobilome ordinations are reported at the class level of ontology, though these results remained consistent when analyzed at the mechanism level (data not shown). For the human dataset **(A)** where patients were parenterally administered a cocktail of antimicrobial drugs for 4 days, the resistome and all studied components of the mobilome (i.e., ICE, plasmids, and prophages) demonstrated a concomitant shift in composition after peak antimicrobial administration (Day 4) which persisted 4 days after peak exposure (Day 8) (ANOSIM *P* < 0.001) and reverted closer to the original composition seen at baseline by Days 42 and 180. In the U.S. beef cattle dataset **(B)** where cohorts of cattle were either given metaphylactic tulathromycin upon arrival or remained untreated, there was no detectable difference in resistome composition between treatment and control groups at baseline and after 11 days of monitoring (ANOSIM *P* > 0.05). On the other hand, the ICE **(II)** and plasmid **(III)** components of the mobilome differed between treated and untreated animals (ANOSIM *P* < 0.05). However, all cattle demonstrated a significant alteration in resistome composition over time, regardless of treatment status (ANOSIM *P* < 0.01). While there was a shift in the plasmidome among all groups over time (ANOSIM *P* < 0.05), the remaining components of the mobilome remained unchanged.

To assess systematic changes in resistome and mobilome composition before and after antimicrobial exposures in the human and cattle trials, NMDS ordination was performed using Hellinger-adjusted Euclidean distances obtained from the transformation of normalized counts of genes at the class and mechanism levels. For the human trial, ordination revealed a markedly altered Days 4 and 8 post-treatment resistome relative to pre-treatment (Day 0), while the resistome reverted closer to its original state by Days 42 (Figure 5, ANOSIM *R* = 0.51, *P* < 0.001; PERMANOVA *F* = 13.78, *P* < 0.001). These results were consistent with the major findings of the original publication. These differences were not significantly impacted by dispersion heterogeneity between groups (*P* = 0.07) at each time point. In the analysis of the mobilome, we detected that ICE (ANOSIM *R* = 0.21, *P* < 0.001; PERMANOVA *F* = 3.50, *P* < 0.001), plasmids (ANOSIM *R* = 0.26, *P* < 0.001; PERMANOVA *F* = 1.68, *P* < 0.01), and prophages (ANOSIM *R* = 0.27, *P* < 0.001; PERMANOVA *F* = 1.52, *P* = 0.02) displayed a concomitant shift in their composition at Days 4 and 8, and reverted to original (Day 0) composition by Day 42. Though beta-dispersion between groups were not significant for plasmids and ICE (*P* > 0.1), differences between timepoints were significantly affected by dispersion for prophage genes (*P* = 0.02). The prophage component therefore is likely explained by both the temporal variation before after antibiotic administration, as well as by inter-group differences in the human fecal samples. Procrustes analysis was performed to assess the degree of correlation between the mobilome and resistome in human fecal samples by superimposing *metaMDS* ordinations of each. Procrustes revealed strong correlation (*P* < 0.001 for all comparisons) in the resistome and mobilome [Pre-treatment: *M*^2^ = 0.32; Peak-treatment (Day 4): *M*^2^ = 0.28; Post-treatment (Day 8–180): *M*^2^ = 0.31] over time.

A similar exploration of the resistome and mobilome composition was undertaken for the cattle dataset. At the mechanism and class levels we detected no differences in the composition of the resistome between feedlot cattle receiving a single dose of tulathromycin and control groups at Day 0 (pre-treatment) and Day 11 (*P* > 0.05). While the ICE component of Day 0 samples was too sparse to analyze, by Day 11, the ICE composition had changed between treated and control groups (ANOSIM *R* = 0.14, *P* = 0.01; PERMANOVA *F* = 1.21, *P* = 0.03). The plasmidome composition differed between treatment and control groups at Day 0 (ANOSIM *R* = 0.11, *P* = 0.02) and Day 11 (ANOSIM *R* = 0.08, *P* = 0.02). The prophage and other MGE components, however, did not differ significantly by treatment group. For comparison between treated and untreated groups there was significant heterogeneity in dispersion for all analyzable components of the resistome (*P* < 0.05), Though the resistome did not differ between treated and control cattle, it shifted significantly over time in both groups of cattle (Treated: ANOSIM *R* = 0.34, *P* < 0.01; PERMANOVA *F* = 8.10, *P* < 0.01; Untreated: ANOSIM *R* = 0.14, *P* = 0.01; PERMANOVA *F* = 6.88, *P* = 0.01), as did the plasmidome (Treated: ANOSIM *R* = 0.26, *P* < 0.001; PERMANOVA *F* = 4.08, *P* < 0.001) Untreated: ANOSIM *R* = 0.17, *P* < 0.01; PERMANOVA *F* = 3.89, *P* < 0.01) (Figure 5). Dispersion was not statistically significantly different (*P* > 0.05). All other components of the mobilome remained unchanged over time in both groups. As with the human dataset, Procrustes analysis was performed on superimposed ordinations of the cattle mobilome and resistome, revealing significant correlation (*P* < 0.001 for all treatment comparisons and *P* < 0.01 for all time comparisons) between treatment groups (Treated *M*^2^ = 0.50; Untreated *M*^2^ = 0.49) and across time [Treated (Day 0): *M*^2^ = 0.30; Treated (Day 11): *M*^2^ = 0.39; Untreated (Day 0): *M*^2^ = 0.19; Untreated (Day 11): *M*^2^ = 0.26].

Based on our Procrustes test results, mobilomes and resistomes are closely correlated, and remain so during antimicrobial exposures. This finding is consistent with previous work on MGEs in microbial genomes in cattle and humans. For instance, evolution of pathogenic *Staphylococcus aureus* has been directly linked to accumulation of MGEs encoding methicillin resistance and virulence (Lindsay, 2010), and MGEs including plasmids, prophages, and integrons have played a pivotal role in the rise of community- and livestock-specific clones with host-adapted AMR profiles (Lindsay et al., 2012).

### Alignment: Interactions Between AMR Genes and MGEs Based on Network Analysis

**FIGURE 6:**
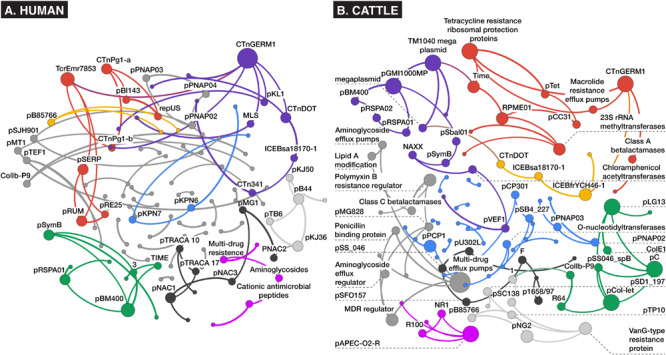
Bayesian networks of **(A)** human and **(B)** cattle metagenomes, depicting nodes corresponding to counts of aligned AMR or MGE accessions at the mechanism level of ontology, and edges (arrows) representing co-occurrence with >70% bootstrap support. Node size and label is proportional to betweenness centrality, and node hue depicts optimal modularity groupings revealed using the Louvain modularity algorithm (Blondel et al., 2008).

Network analysis of alignment-based count data revealed relatively sparse networks for both the human and cattle datasets, which consisted of 113 nodes/86 edges and 102 nodes/95 edges, respectively, with most nodes containing between one and six connections (Figure 6). Both dataset’s networks were dominated by a large density of intra-mobilome edges as a proportion of all edges (human: 74% plasmid-plasmid, 10% ICE-ICE, 6% plasmid-ICE; cattle: 57% plasmid-plasmid, 4% ICE-ICE, 2% plasmid-ICE) (Supplementary Datasheets 3, 4). Edges between AMR groups and MGEs comprised ∼19% of total edges in the cattle network, and only ∼2% of the human network. Within bovine fecal samples, the ICE family of CTnGERM1, transposon TcrEmr7853, and conjugative transposon family CTnDOT, tended to co-occur with AMR mechanisms involving macrolide efflux systems, erythromycin-related methyltransferase systems, and tetracycline ribosomal protection proteins, respectively. These findings are consistent with previous genomic and functional characterizations of these MGE families, which have confirmed the presence of modular regions containing accessory cassettes encoding resistance to macrolides (Cooper et al., 1996; Whittle et al., 2001; Wang et al., 2003, 2005) and tetracyclines (Wang et al., 2003; Wozniak and Waldor, 2010; Toleman and Walsh, 2011; Waters et al., 2013), respectively. The fact that network analysis revealed these ICE-AMR connections is especially noteworthy given that ICE alignments were such a small proportion of all mobilome alignments within the cattle dataset (Figure 2); this suggests that network analysis can uncover potentially meaningful biological relationships even for relatively low-abundance metagenomic features. Furthermore, while network edges between AMR mechanisms and MGE families represent putative and largely correlative associations, it is potentially noteworthy that the AMR mechanisms identified as sharing edges with MGEs correspond to antimicrobials that are most commonly administered in beef cattle, especially in feedlot settings (USDA, 2011). The presence of these resistance mechanisms on MGEs, therefore, may be an evolutionary response of feedlot-associated bacterial populations to extant evolutionary pressures. On the other hand, the cattle fecal samples also contained apparent linkages between aminoglycoside and glycopeptide AMR mechanisms and a variety of plasmids including *pKPN3* and *pSC138* (Figure 6). The potential mobilization of these AMR mechanisms is not easily explained by either common cattle production antimicrobial drug practices or the specific study population analyzed, as glycopeptide antimicrobials are prohibited from use in North American cattle (CFR 21, 1997), and aminoglycosides are rarely used due to prolonged withdrawal periods (Gehring et al., 2005). Given the preponderance of plasmidome alignments in the cattle dataset (Figure 2), these edges may represent an important component of cattle-adapted microbiomes in the absence of specific antimicrobial drug pressures. Interestingly, resistance mechanisms to glycopeptides were sparsely identified using the alignment approach (Figure 4). In the cattle dataset, 38 and 20 unique gene groups were identified by *MetaCompare* and metaSPAdes+BLAST, respectively, whereas only a single unique gene group was identified by alignment. However, a recent study systematically profiling AMR genes of beef and dairy cow rumens, identified that glycopeptide resistance genes are among the most common components of the ruminal resistome (Sabino et al., 2019). Therefore, it is likely that the network based on alignment underestimates glycopeptide-MGE connections.

Contrarily, in the human study, where patients were administered meropenem (carbapenem), gentamycin (aminoglycoside), and vancomycin (glycopeptide), there was little evidence of co-occurrence of MGEs with resistance mechanisms for these classes of drugs. Instead, an edge was identified between a macrolide-lincosamide-streptogramin (MLS) mechanism and the CTnGERM1 ICE family. This was the only predicted AMR-MGE edge identified in the human dataset. This association may have been incidental given that macrolide resistance genes are among the most relatively abundant genes in the human gut resistome (van Schaik, 2015), and some (e.g., *ermB/F/G*) are considered among the most promiscuous in the human resistome (Salyers et al., 2004). The lack of AMR-MGE edges within the human fecal samples is difficult to explain, particularly given the multi-drug treatment administered, which theoretically should have placed intense selective pressure on the bacteria to promote HGT of relevant AMR mechanisms.

Based on human clinical isolates, though MGE-mediated transfer of AMR genes occurs more commonly among related microbial taxa, resistance conjugation between distantly related bacteria has been extensively documented (van Schaik, 2015). Therefore, results of co-occurrence networks must be interpreted with a great deal of caution, as “statistical colocalization” is likely necessary but not sufficient to predict mobilization of genes, and any hazard associated with identified co-occurrences must be assessed in light of what is known regarding the history of antimicrobial drug exposures, clinical history and disease status, gut microbial ecology, genetic context of the mobile genes and resistance genes, as well as host and environmental factors.

### Assembly: AMR-MGE Co-occurrence Results Differ Based on Assembly Method

To identify potential genomic colocalizations between AMR genes and MGEs, we undertook graph-based assembly of a subset of samples in each dataset. Because assembler choice can profoundly impact assembly statistics for metagenomic data (Vollmers et al., 2017) we compared two common metagenomic assemblers, IDBA-UD (as implemented in *MetaCompare*) and metaSPAdes. On average across both datasets, metaSPAdes produced 323,000 contigs per sample compared to 271,000 for IDBA-UD assembler of the *MetaCompare* pipeline. The N50 for metaSPAdes assemblies was significantly higher than for IDBA-UD assemblies, even when controlling for dataset (mean 6,260 bp versus 1,702 bp, *P* = 0.02), suggesting that, on average, metaSPAdes produced significantly longer contigs than IDBA-UD (Supplementary Datasheet 6).

Strikingly, while *MetaCompare* revealed numerous contigs containing *either* MGE *or* AMR gene accessions, none of these contigs contained *both* (Supplementary Datasheet 7). Therefore, the resulting risk score calculated by the *MetaCompare* pipeline was not informative in terms of mobilization potential of the identified AMR genes. On the other hand, metaSPAdes and subsequent BLAST revealed numerous contigs containing at least one each of AMR and MGE genes (Table 2). In the human and cattle datasets, the number of AMR- and MGE-containing contigs per sample ranged from 10 to 65 and 61 to 93, respectively. Therefore, the metaSPAdes+BLAST approach identified more colocalizations than *MetaCompare*. This could be explained partially by the fact that the metaSPAdes assemblies produced significantly longer N50 than IDBA-UD. Indeed, the mean N50 of metaSPAdes was long enough to easily contain both a full-length AMR gene and either a partial or full-length MGE; by comparison, the mean N50 of IDBA-UD was barely long enough to contain the full length of most AMR genes.

**TABLE 2:** Summary of assembly results indicating the abundance of sample-level contigs, co-occurring and non-co-occurring resistome and mobilome accessions identified on contigs, and results of assembly based colocalization analysis reported as a Mobility Index for human and cattle datasets (MI = proportion of all contigs within a sample containing at least one resistance gene flanked by at least one locus with a MGE residue, relative to all contigs with resistance).

**Dataset**	**Sample**	**Individual**	**Treatment**	**Time**	**nContigs**	**nAMR**	**nMGE**	**nAMR&MGE**	**nAMR/nContigs**	**nMGE/nContigs**	**Mobility index (MI)**	**Overlap adjusted MI**
**Human**												
	15	A	Pre-MGV	Day 0	3,730	209	11,889	12,098	0.06	3.19	0.25	0.02
	18	A	MGV Final	Day 4	408	29	1,448	1,477	0.07	3.55	0.00	–
	19	A	Post-MGV	Day 8	1,605	1,335	25,090	26,425	0.83	15.63	0.51	0.20
	17	A	Post-MGV	Day 42	2,566	187	9,123	9,310	0.07	3.56	0.47	0.03
	16	A	Post-MGV	Day 180	3,103	298	9,645	9,943	0.10	3.11	0.50	0.04
	20	B	Pre-MGV	Day 0	2,758	650	12,877	13,527	0.24	4.67	0.21	0.02
	23	B	MGV Final	Day 4	272	185	2,838	3,023	0.68	10.43	0.78	0.76
	24	B	Post-MGV	Day 8	1,338	220	7,553	7,773	0.16	5.64	0.48	0.29
	22	B	Post-MGV	Day 42	1,376	85	4,259	4,344	0.06	3.10	0.40	0.08
	21	B	Post-MGV	Day 180	2,255	609	8,961	9,570	0.27	3.97	0.21	0.03
**Cattle**												
	11p2	A	Tulathromycin	Day 11	5,663	626	20,321	20,947	0.11	3.59	0.25	0.02
	102p2	B	Tulathromycin	Day 11	5,858	478	16,955	17,433	0.08	2.89	0.43	0.03
	103	C	Tulathromycin	Day 0	11,708	657	38,848	39,505	0.06	3.32	0.39	0.06
	103p2	C	Tulathromycin	Day 11	7,169	632	21,326	21,958	0.09	2.97	0.41	0.01
	130	D	Tulathromycin	Day 0	8,543	572	23,845	24,417	0.07	2.79	0.36	0.01
	130p2	D	Tulathromycin	Day 11	7,816	782	21,129	21,911	0.10	2.70	0.32	0.01
	155p2	E	Tulathromycin	Day 11	5,756	589	17,457	18,046	0.10	3.03	0.36	0.01
	156	F	Tulathromycin	Day 0	6,264	544	20,440	20,984	0.09	3.26	0.32	0.04
	156p2	F	Tulathromycin	Day 11	7,599	1,050	25,094	26,144	0.14	3.30	0.26	0.01
	158	G	Tulathromycin	Day 0	10,779	802	35,010	35,812	0.07	3.25	0.28	0.02
	164	H	Tulathromycin	Day 0	9,250	657	29,898	30,555	0.07	3.23	0.32	0.03
	164p2	H	Tulathromycin	Day 11	5,643	542	17,191	17,733	0.10	3.05	0.38	0.02
	208	I	Untreated	Day 0	6,488	484	20,762	21,246	0.07	3.20	0.31	0.01
	208p2	I	Untreated	Day 11	9,377	784	26,870	27,654	0.08	2.87	0.31	0.01
	216p2	J	Untreated	Day 11	6,757	752	23,309	24,061	0.11	3.45	0.28	0.01
	220	K	Untreated	Day 0	6,637	491	22,856	23,347	0.07	3.44	0.30	0.01

*MIs are used as a means to compare the relative abundance of the putatively “mobile” fraction of resistance genes in samples.*

Though a number of studies focusing on interrogating the potential for components of microbial resistomes to be mobilized in various contexts, there are currently no widely accepted methods used to quantify mobility potential, HGT-likelihood, or any objective measure of resistance transmission risk. This is especially the case in the analysis of mobility based on metagenomic assembly data. Based on similar approaches used in this field, we reported the mobility index (MI) for each sample which reflects the total fraction of contigs with at least one AMR region that is also flanked by at least one nearby MGE region, relative to all AMR-containing contigs within the sample. In critically evaluating the colocalization identified by metaSPAdes+BLAST, we noted many instances in which the start and stop alignment positions for the AMR gene overlapped substantially with the start and stop positions of the MGE. Upon closer inspection, it became apparent that the MGE reference databases contained MGE accessions that included accessory or cargo sequences, including AMR genes. This “identity contamination” in current-day MGE reference databases presents an inherent impediment to colocalization analysis of assembled contigs, and additional work must be done to correct for co-occurrence misclassification. For instance, a region of a contig that maps to the *TetM* accession in an AMR database might also map to the transcriptional regulator region of the *Tn916* conjugative element within an MGE database because this region contains a *TetM* complex. Therefore, the contig would be potentially erroneously classified as containing both *TetM* and *Tn916*.

To avoid such examples of misclassification and obtain a more accurate estimate of the number of contigs with colocalizations, we performed overlap analysis and calculated both a raw and an adjusted Mobility Index (MI), as described in section “Materials and Methods.” The disparity between raw and overlap-adjusted MIs reached 1–2 orders of magnitude (Table 2), highlighting the extent of misclassification due to overlap. The MI results also revealed patterns of mobilome-resistome dynamics that were not detected using alignment-based or *MetaCompare* results. For example, for one human subject, adjusted MI was at ∼2% prior to antimicrobial exposures, but then increased ∼75% and in all cases mobility potential remained elevated at > 20% at 4 days after receiving the last dose. The overlap-adjusted MIs decreased to roughly baseline by Days 42 and 180 of the total study period. On the other hand, in the cattle dataset, mobility potential among those animals exposed to tulathromycin metaphylaxis did not significantly differ from control animals. Moreover, across all animals, mobility potential did not significantly differ over time. Though not a primary focus of the study, it is noteworthy that human samples at Days 0 and 180 had a generally similar adjusted MI as that seen across all cattle samples and that the mean adjusted MI across all human samples associated with recent antimicrobial exposure was 10-fold greater than adjusted MIs of cattle samples (Student’s *t* = 2.405, *P* = 0.01). This difference in mobility index among humans and animals when exposed to antimicrobials should be further explored in future work involving samples appropriately statistically powered for such analyses. Though we are noting differences in MI between human and animal trials to illustrate the way in which mobility analysis of resistomes can lend itself to hazard stratification and prioritization, it should be noted that a number of factors hinder a valid comparison of resistome mobility potential between the two datasets. Both studies collected a relatively small number of samples from a limited population set; both populations were administered different antimicrobial regimens to achieve different clinical endpoints; and metagenomes from both studies were collected, processed, and sequenced using different laboratory, instrumental, and computational protocols. Therefore, a robust comparison of the resistomes in these two datasets is not supported.

Discovery of AMR genes in genomic proximity to MGEs within contigs is considered evidence of potential mobility via HGT (Bengtsson-Palme et al., 2014; Oh et al., 2018) and a number of studies have reported on the localization of both AMR sequences and MGEs in close proximity on the same stretch of contig. Our results demonstrate that colocalization based on assembly and subsequent alignment to existing databases is potentially fraught with both incompleteness and inaccuracy. Incompleteness stems from the fact that colocalization necessitates the reconstruction of contigs with at *least* several thousand base-pairs. As demonstrated, even metagenomic-specific assemblers produce relatively fragmented assemblies with small N50 values; the likelihood of reconstructing MGEs and AMR “cargo” genes, especially those in excess of ∼1000 bp, is low given this fact. We demonstrated the importance of high-quality assemblies by showing that, although *MetaCompare* identified more unique AMR gene groups than metaSPAdes+BLAST (Figure 3), it failed to identify a single contig with both an AMR gene and an MGE. The strongest hypothesis for this is that the assemblies produced by IDBA-UD within *MetaCompare* contained a significantly shorter N50 than the assemblies produced by metaSPAdes. Our findings are consistent with previous work comparing the metaSPAdes assembler with other assemblers in gene identification (Nurk et al., 2017; Vollmers et al., 2017). This difference in N50 is critical for co-occurrence analysis, as longer stretches of DNA are more likely to produce sufficient sequence space to identify multiple genes, inherent to colocalization. While the metaSPAdes+BLAST results did contain numerous contigs with both AMR genes and MGEs, closer inspection of these colocalizations revealed a major source of colocalization inaccuracy, namely sequence homology between numerous accessions within the AMR and MGE reference databases. Analysis of BLAST outputs of contigs mapped to AMR and especially MGE databases, revealed many high-confidence (e < 1 × 10^–50^) hits to multiple families of ICE, plasmid, prophage and other MGEs to the same regions of a given contig (Supplementary Datasheet 8). For example, for the human dataset, metaSPAdes identified an average of 16 unique MGE groups per contig, and 5 AMR accessions per contig. *MetaCompare* yielded a similarly large contig-level richness, though because *MetaCompare* utilizes a “gene fraction” cut-off for mapping contigs to ACLAME, the MGE contig-level richness was smaller relative to that of metaSPAdes. Nevertheless, the fact that multiple hits to genetically distinct families of MGEs were found on the same contigs highlights the fact that current AMR and MGE databases are plagued by substantial intra-mobilome/-resistome sequence homology, which likely precludes confident identification of AMR and MGE features, particularly at resolved levels of the ontology. This database problem was most starkly highlighted by our overlap analysis, which showed that even AMR database accessions can share large stretches of sequence homology with current MGE database accessions.

## Conclusion

Resistome-mobilome colocalization analysis is complex and still in its infancy. While we attempted to review the advantages and pitfalls of common colocalization approaches, it was not possible to definitively identify which approach yielded the most accurate answer. Such an analysis would require diverse metagenomic samples with “known truth.” However, our results strongly suggest that colocalization results should be extensively tested for robustness in the face of changes in bioinformatic approach. In other words, hypothesize that validity of conclusions is most likely to be appreciable if findings of the various alignment-based and assembly-based approaches are considered in tandem. For example, for human fecal samples, ordination analysis seems to reveal that in general, while resistomes and mobilomes are both dynamic in the face of antimicrobial administration and over time, the resistome-mobilome compartments respond in a monotone fashion. Moreover, network analysis reveals that overall, predictive co-occurrence of major resistance and mobile element clusters are rare. Further, colocalization on the basis of assembly suggests that though a greater fraction of contigs containing both resistance and mobile genetic determinants are found in samples collected shortly after parenteral administration of antimicrobial drugs, and thus may be a source of greater opportunity for resistance mobilization, this was not permanent, as co-occurrence frequency returned to baseline after ∼11 weeks of last antibiotic course. For fecal samples collected from cattle entering a beef feedlot and were exposed to metaphylactic levels of a macrolide antimicrobial, ordination reveals a rather static resistome and mobilome in the face of antimicrobial exposure. Though Bayesian networks predicted numerous significant clusters of resistance and mobile elements across all samples, overall frequency of co-occurrence of AMR genes and MGEs in assembled contigs did not change as a function of antimicrobial exposure.

Many studies that characterize resistomes imply that the identification of AMR genes is a putative indication of public health risk or hazard, whereby resistance factors can be acquired from their ecological context by pathogens and therefore become more recalcitrant in the face of standard medical therapy or antimicrobial intervention. However, *de facto* detection and quantification of resistomes in a sample is not sufficient, because AMR genes are not “risk-equal.” For example, the presence of an AMR gene that confers resistance to a last-resort antimicrobial being carried by a pathogen likely has higher risk than the same AMR gene within a benign bacterium. Likewise, an AMR gene located on a promiscuous plasmid likely carries higher mobility and health risk than an AMR gene located within a chromosome. These examples illustrate the need to understand the metagenomic context of AMR genes. In response to this realization, many researchers are pairing resistome analyses with an analysis of the “mobilome.” As resistome-mobilome analyses are relatively new, utilizing human clinical and agricultural datasets, we critique the current state of commonly used bioinformatic and statistical approaches to reveal co-occurrence of resistomes and mobilomes, as well as current methods for estimating the mobilizability of AMR genes within a microbiome. Our results demonstrate that colocalization approaches are limited by technical challenges inherent in current reference databases, analytical pipelines, and metagenomic sequence data. Therefore, alignment-based and assembly-based methodologies often yield insufficient, incomplete, inaccurate, and/or conflicting information.

At this time, advancements are being made to address some of these deficiencies. For example, in hopes of increasing mobilome and resistome detection sensitivity, new laboratory methods are being proposed, including the use of targeted resistome and mobilome enrichment (Noyes et al., 2017; Guitor et al., 2019). Long-read and hybrid sequencing techniques may eventually accommodate the throughput required for diverse, high bacterial-load metagenomic samples, in which case bioinformatic techniques for detection of AMR genes and MGEs would become much more straightforward (Arango-Argoty et al., 2018), although we point out that database inconsistences would remain a barrier. Additionally, computational approaches are evolving in an effort to support more complete and accurate resistome-mobilome colocalization from short-read metagenomic data (Roodgar et al., 2019; Stalder et al., 2019). Finally and most recently, new approaches have been proposed to increase sensitivity of MGE and AMR discovery and classification (Jiang et al., 2019; Durrant et al., 2020). Though we have expressly highlighted the pervasive issue of cargo sequences in MGE databases, a similar problem likely exists in AMR databases as well. For example, within MEGARes, some accessions annotated as AMR genes may actually contain accessory, cargo, or flanking regions that are not specifically related to AMR genes themselves. This study highlights the use of existing databases and tools for resistome mobility analysis and is therefore not focused on identifying and untangling discrepant sequences in AMR gene and MGE accessions. Given the state of current databases and the findings presented here, we strongly urge investigators to integrate database validation tools as a standard component of metagenomic analysis. For AMR databases in particular, recent pipelines such as DeepARG (Arango-Argoty et al., 2018) and ARGDIT (Chiu and Ong, 2019) have been developed to improve accession validity, consensus, and accuracy. Use of such tools may help to circumvent some of the challenges posed by the structure and content of current reference databases. In addition to these database validation tools, there is continued innovation around statistical, bioinformatic, sequencing and technical tools for improving the sensitivity and applicability of resistome-mobilome analysis. Such advancements should be continuously integrated into resistome-mobilome analyses.

## Data Availability Statement

The datasets generated for this study can be found in the ENA (EMBL-EBI) ERP022986 (Palleja et al., 2018), SRA (NCBI) PRJNA309291 (Doster et al., 2018).

## Author Contributions

NN and IS designed this study and provided oversight for all other aspects of the study. IS, NN, KM, CB, and CD oversaw and performed bioinformatic analysis, while IS and NN performed all related statistical analysis. KM and IS performed the colocalization analysis of assemblies. IS, NN, and CB contributed equally to drafting and refinement of the manuscript. All authors approved the final manuscript prior to submission.

## Conflict of Interest

The authors declare that the research was conducted in the absence of any commercial or financial relationships that could be construed as a potential conflict of interest.

## Footnotes

1 https://doi.org/10.13020/jw1t-8n48

## Supplementary Material

DATASHEET S1 Sample metadata, sequence quality scoring and filtering, and raw AMR/MGE gene accession counts for the cattle metagenomic dataset (Doster et al., 2018).

DATASHEET S2 Sample metadata, sequence quality scoring and filtering, and raw AMR/MGE gene accession counts for the human metagenomic dataset (Palleja et al., 2018).

DATASHEET S3 Directed list of edges of the Bayesian Network of AMR and MGE accessions for the cattle metagenomic dataset.

DATASHEET S4 Directed list of edges of the Bayesian Network of AMR and MGE accessions for the human metagenomic dataset.

DATASHEET S5 Heatmap resistance gene mechanism names corresponding to resistance gene class (across rows).

DATASHEET S6 *De novo* assembly quality and performance parameter summary for IDBA-UD (*MetaCompare*) and metaSPAdes assemblies for all metagenomic datasets.

DATASHEET S7 Default summary output of resistome-mobilome co-occurrence analysis and mobility risk score using the *MetaCompare* pipeline.

DATASHEET S8 Summary of sample-level and contig-level AMR and MGE gene richness for assembled metagenomes using either *MetaCompare* or metaSPAdes for all metagenomic datasets.
